# Metabolite Profiles of the Cerebrospinal Fluid in Neurosyphilis Patients Determined by Untargeted Metabolomics Analysis

**DOI:** 10.3389/fnins.2019.00150

**Published:** 2019-02-26

**Authors:** Li-Li Liu, Yong Lin, Wei Chen, Man-Li Tong, Xi Luo, Li-Rong Lin, Hui-Lin Zhang, Jiang-Hua Yan, Jian-Jun Niu, Tian-Ci Yang

**Affiliations:** ^1^Center of Clinical Laboratory, Zhongshan Hospital, School of Medicine, Xiamen University, Xiamen, China; ^2^Institute of Infectious Disease, School of Medicine, Xiamen University, Xiamen, China; ^3^Shanghai Applied Protein Technology Co., Ltd., Shanghai, China; ^4^Cancer Research Center, School of Medicine, Xiamen University, Xiamen, China

**Keywords:** untargeted metabolomics, neurosyphilis, cerebrospinal fluid, *Treponema pallidum*, metabolites

## Abstract

The mechanism underlying the stealth property of neurosyphilis is still unclear. Global metabolomics analysis can provide substantial information on energy metabolism, physiology and possible diagnostic biomarkers and intervention strategies for pathogens. To gain better understanding of the metabolic mechanism of neurosyphilis, we conducted an untargeted metabolomics analysis of cerebrospinal fluid (CSF) from 18 neurosyphilis patients and an identical number of syphilis/non-neurosyphilis patients and syphilis-free patients using the Agilent, 1290 Infinity LC system. The raw data were normalized and subjected to subsequent statistical analysis by MetaboAnalyst 4.0. Metabolites with a variable importance in projection (VIP) greater than one were validated by Student’s *T*-test. A total of 1,808 molecular features were extracted from each sample using XCMS software, and the peak intensity of each feature was obtained. Partial-least squares discrimination analysis provided satisfactory separation by comparing neurosyphilis, syphilis/non-neurosyphilis and syphilis-free patients. A similar trend was obtained in the hierarchical clustering analysis. Furthermore, several metabolites were identified as significantly different by Student’s *T*-test, including L-gulono-gamma-lactone, D-mannose, N-acetyl-L-tyrosine, hypoxanthine, and *S*-methyl-5′-thioadenosine. Notably, 87.369-fold and 7.492-fold changes of N-acetyl-L-tyrosine were observed in neurosyphilis patients compared with syphilis/non-neurosyphilis patients and syphilis-free patients. These differential metabolites are involved in overlapping pathways, including fructose and mannose metabolism, lysosomes, ABC transporters, and galactose metabolism. Several significantly expressed metabolites were identified in CSF from neurosyphilis patients, including L-gulono-gamma-lactone, D-mannose, N-acetyl-L-tyrosine, and hypoxanthine. These differential metabolites could potentially improve neurosyphilis diagnostics in the future. The role of these differential metabolites in the development of neurosyphilis deserves further exploration.

## Introduction

Syphilis is a common sexually transmitted disease worldwide, and the causative pathogen is the spirochete *Treponema pallidum* (Tong et al., 2013; Giacani and Lukehart, 2014). In China, the incidence of syphilis has demonstrated an increasing trend, with an annual growth percentage of 16.3% (Yang et al., 2017), and ranks first among 45 notifiable diseases (Yang et al., 2017). Although the significant growth in syphilis incidence in China can be partly attributed to the implementation of enhanced coverage and the completeness of nationwide, web-based and real-time reporting systems, syphilis is a sexually transmitted disease with a severe public health impact and should not be neglected.

Neurosyphilis refers to *T. pallidum* infection in the central nervous system, and it can occur at any stage of syphilis (Liu et al., 2017). In general, the most common form of neurosyphilis currently diagnosed in clinical practice is asymptomatic. The stealth property of *T. pallidum* is responsible for the obstacles currently encountered in the treatment and prevention of syphilis. To date, the mechanism underlying the stealth property has not yet been fully elucidated. Previous studies have proposed that the slow replication cycle of *T. pallidum* *in vivo* may be one of the reasons for the diminished immune response and clinical symptoms after infection (Wicher et al., 2000; Deka et al., 2013). Given a plentiful supply of glucose in the blood and interstitial fluids, *T. pallidum* requires approximately 33 to 44 h to double in number (Edmondson et al., 2018). The limited energy metabolic capacity of *T. pallidum* is responsible for the slow replication cycle and inability to survive outside mammalian cells of this pathogen (Deka et al., 2015). With the completion of whole genome sequencing of *T. pallidum*, the total size of 1.14 Mb and 1041 predicted open reading frames have been identified (Fraser et al., 1998). Compared with that of other bacterial pathogens, the genome of *T. pallidum* is several times smaller, and the absence of pathways related to the tricarboxylic acid cycle and components of oxidative phosphorylation in the limited genome have led to the major dependence on the host environment to perform the necessary biosynthetic functions (Tong et al., 2017). Endogenous metabolites are an excellent reflection of the metabolic process of pathogens in hosts.

Owing to the high sensitivity and specificity of state of the art ultra-high-performance liquid chromatography/quadrupole-time-of-flight-mass spectrometry (UHPLC-Q-TOF/MS), even tiny variations in metabolites can be detected (Zhu et al., 2016). Global metabolomics analysis has been widely applied in investigations of pathogen-related diseases (Wang et al., 2016; Du et al., 2017). For instance, a study conducted among 30 pairs of HCV-positive patients and healthy controls revealed several differentially excreted metabolites in urine, and after enrichment analysis, enhanced aldose reductase activity was identified as a hallmark among HCV patients (Semmo et al., 2015). Such studies are capable of providing substantial information on energy metabolism, physiology and possible diagnostic biomarkers and intervention strategies for the pathogen (Chen et al., 2017). To date, no publications on the metabolomics analysis of syphilis patients have been reported. To gain a better understanding of the metabolic changes of neurosyphilis patients, we conducted an untargeted metabolomics analysis on cerebrospinal fluid (CSF) samples collected from neurosyphilis patients, syphilis/non-neurosyphilis patients and syphilis-free patients. By conducting this study, we attempted to identify a few hallmark metabolites in the CSF among neurosyphilis patients and consequently reveal the physiology and possible diagnostic biomarkers in neurosyphilis for further investigation.

## Materials and Methods

### Study Participants

Consecutive neurosyphilis patients (Group-1) were recruited from department of Neurology Zhongshan Hospital, Xiamen University between July 2017 and December 2017, with 18 cases. Considering sex- and age-matching with the neurosyphilis patients, syphilis/non-neurosyphilis patients (Group-2) and syphilis-free patients (Group-3) were selected from the department of Neurology department of Neurosurgery in the same period of the time with 18 cases in each group. According to the guidelines of the European Center for Disease Prevention and Control, the diagnosis of syphilis was established in the present study by employing treponemal tests, including chemiluminescence immunoassays and *T. pallidum* particle agglutination (TPPA) (French et al., 2009). The diagnostic criteria of neurosyphilis in our study complied with the guidelines of the Centers for Disease Control in America and Europe (Janier et al., 2014; Workowski and Bolan, 2015) as described in our previous study (Xiao et al., 2017a). Briefly, neurosyphilis was defined based on positive treponemal test results and one or more of the following findings: (i) positive CSF Venereal Disease Research Laboratory (VDRL) and/or rapid plasma reagin (RPR) tests; (ii) positive TPPA assay and elevated leukocyte count (>10 cells/μL) in CSF; and (iii) elevated CSF protein concentration (>500 mg/L) and/or leukocyte count (>10 cells/μL) in the absence of other known causes of these abnormalities and clinical symptoms or signs consistent with neurosyphilis without other known causes for these clinical abnormalities. Syphilis/non-neurosyphilis patients were diagnosed if (i) they were seropositive for treponemal tests, including chemiluminescence immunoassays and TPPA; and (ii) they had negative results on the CSF VDRL/RPR, CSF TPPA, and CSF fluorescent treponemal antibody absorption assays, without CSF pleocytosis and elevated CSF protein levels, and did not exhibit any characteristic symptoms or signs of neurosyphilis (Xiao et al., 2017a). Syphilis-free patients were recruited by the neurosurgery department, and these patients were hospitalized due to acute trauma which subjected to lumber puncture to exclude the possibility of infectious diseases. Potential participants were included in syphilis-free group if they showed all negative results in serum RPR, serum TPPA, CSF RPR, and CSF TPPA.

### Ethics Statement

This study was carried out in accordance with the recommendations of the Institutional Ethics Committee of Zhongshan Hospital, Medical College of Xiamen University with written informed consent from all subjects. All subjects gave written informed consent in accordance with the national legislation and the Declaration of Helsinki. The protocol was approved by the Institutional Ethics Committee of Zhongshan Hospital, Medical College of Xiamen University.

### Sample Preparation

Approximately 2 mL of CSF was collected from each patient by conducting lumbar puncture from neurosyphilis patients and an identical number of syphilis/non-neurosyphilis patients and syphilis-free patients. After collection, the samples were immediately placed on ice for transportation; processed for syphilitic serological tests, CSF protein and leucocyte counted within 6 h of obtaining the CSF; and then stored at -80°C prior to further processing for UHPLC-Q-TOF/MS analysis. Before analysis, the samples were thawed at room temperature. Then, 100 μL of sample was transferred to EP tubes and mixed with 400 μL of methanol/acetonitrile (1:1, v/v). The tubes were vortexed for 30 s, incubated for 10 min at -20°C, and then centrifuged at 14000 g for 15 min at 4°C. The supernatants were collected and dried with nitrogen, and then, the lyophilized powder was stored at -80°C prior to analysis. Lyophilized samples were reconstituted by dissolving in 100 μL of solvent mixture containing water/acetonitrile (5:5, v/v). The samples were vortexed for 1 min and centrifuged at 14000 g for 15 min at 4°C. The supernatants were subjected to UHPLCQ-TOF/MS analysis. In parallel to the preparation of the test samples, pooled quality control (QC) samples were prepared by mixing equal amounts (30 μL) of each sample. The QC samples were utilized to monitor the LC-MS response in real time (Ma et al., 2017).

### Laboratory Tests

The syphilitic serological tests for each sample were performed using RPR (InTec, Xiamen, China), Boson chemiluminescence immunoassays (Boson Biotechnology Co., Ltd., Xiamen, China), and TPPA (Fujirebio, Tokyo, Japan) tests according to the manufacturer’s instructions and as previously reported (Xiao et al., 2017b). The protein in the CSF samples was measured using a Roche-Hitachi Modular P800 Analyzer (Roche Diagnostics, F. Hoffmann-La Roche, Ltd., Basel, Switzerland). The CSF leukocyte count was measured using an Automatic Blood Cell XE5000 Analyzer (Sysmex International Reagents, Co., Ltd., Japan).

### UHPLC-Q-TOF/MS Analysis

Metabolic profiling of CSF samples was performed on an Agilent, 1290 Infinity LC system (Agilent Technologies, Santa Clara, California, CA, United States) coupled with an AB SCIEX Triple TOF 6600 System (AB SCIEX, Framingham, MA, United States) (Wang et al., 2017). Chromatographic separation was conducted on ACQUITY HSS T3 1.8 μm (2.1 × 100 mm) columns for both positive and negative models. The column temperature was set at 25°C for operation. The mobile phases of 0.1% formic acid in water (A) and 0.1% formic acid in acetonitrile (B) were used in positive ionization mode, while 0.5 mM ammonium fluoride in water (C) and acetonitrile (D) were used in negative ionization mode. In the positive (negative) model, the elution gradient initially started with 1% B (D) for 1 min, linearly increased to 100% B (D) at 8 min, was maintained for 2 min, and then returned to 1% B (D) for approximately 2 min of equilibrium. The delivery flow rate was 300 μL/min, and a 2 μL aliquot of each sample was injected onto the column. UHPLC-Q-TOF/MS was performed on both ionization modes. Electrospray ionization source conditions on Triple TOF were set as follows: the pressure for ion source gas 1 was 40 psi, and the pressure for ion source gas 2 was 60 psi; the pressure for curtain gas was 30 psi; source temperature, 650°C; ionspray voltage floating, 5000 V (+) and -4500 V (-). Information-dependent acquisition, an artificial intelligence-based product ion scan mode, was used to detect and identify MS/MS spectra. The parameters were set as follows: declustering potential, 60 V (+) and -60 V (-); collision energy, 50 V (+) and -20 V (-); exclude isotopes within 4 Da, candidate ions to monitor per cycle: 10. The analysis process was conducted with the assistance of Applied Protein Technology (Shanghai, China).

### Data Analysis

The raw data generated by UPLC-Q-TOF/MS were converted into mzML format files using the Proteo Wizard MS converter tool and then subjected to data processing using XCMS online software (https://xcmsonline.scripps.edu/landing_page.php?pgcontent=mainPage). The non-linear alignment in the time domain, automatic integration, and extraction of the peak intensities were completed by XCMS, with default parameter settings. The data were subsequently processed using XCMS for peak alignment and data filtering. MetaboAnalyst 4.0 (http://www.metaboanalyst.ca) was employed for the statistical analysis (Chong et al., 2018). Principal component analysis (PCA) is initially applied to obtain an overview of the data, especially for the examination of QC data. Partial-least squares discrimination analysis (PLS-DA) was conducted as a supervised method to identify the important variables with discriminative power. PLS-DA models were validated based on the multiple correlation coefficient (R2), after that, we applied cross-validation on this R2 to calculate the cross-validated R2 (Q2); and permutation tests by applying 2000 iterations (*P* < 0.001). The significance of the biomarkers was evaluated by calculating the variable importance in projection (VIP) score (>1) from the PLS-DA model. For the univariate analysis, specific biomarkers were compared among the neurosyphilis, syphilis/non-neurosyphilis, and syphilis-free groups by employing Student’s *T*-tests. Among the metabolites with a VIP greater than 1, those with a *P*-value ranging from 0.05 to 0.10 were considered differential metabolites, while a *P*-value less than 0.05 was considered significant (Luo et al., 2017). The heat map was created by using the embedded module of MetaboAnalyst 4.0, to be more specific, we applied Euclidean distance measure and ward clustering algorithm in creating the heat map. Meanwhile, based on the differentially expressed metabolites, we compared the groups, and KEGG pathway (http://www.genome.jp/kegg/) analysis was conducted to investigate the metabolomic pathways affected by *T. pallidum* infection. Briefly, the enrichment level of each metabolomic pathway was calculated by Fisher’s exact test, and a *P*-value less than 0.05 was considered statistically significant.

## Results

### Baseline Information of Study Participants

The demographic characteristics of all study participants are presented in Table 1. The three groups demonstrated a roughly equal distribution in gender. The average age of the neurosyphilis patients in the study was 56.2 years, and there were 10 males (55.6%) and 8 females (44.5%). The average age of the syphilis/non-neurosyphilis patients was 51.8 years, and there were 9 males (50.0%) and 9 females (50.0%). The average age of the syphilis-free patients was 50.7 years, and there were 9 males (50.0%) and 9 females (50.0%). For CSF RPR, 88.9% of neurosyphilis patients had positive results. We obtained all positive TPPA results in neurosyphilis cases.

**Table 1:** Clinical information of study participants.

	Syphilis/Neurosyphilis	Syphilis-non-neurosyphilis	free	
	patients	patients	patients	
Variable	(*n* = 18)	(*n* = 18)	(*n* = 18)	*P*-value
Age (Years)	58.5(21)	58.5(11.8)	53.0(27)	0.747
Gender				
Male n(%)	10 (55.6)	9 (50.0)	9 (50.0)	0.929
Female n(%)	8 (44.5)	9 (50.0)	9 (50.0)	
CSF RPR				
Negative n(%)	2 (11.1)	18 (100.0)	18 (100.0)	
Positive n(%)	16 (88.9)	0 (0.0)	0 (0.0)	
CSF TPPA				
Negative n(%)	0 (0.0)	18 (100.0)	18 (100.0)	
Positive n(%)	18 (100.0)	0 (0.0)	0 (0.0)	


*CSF, cerebrospinal fluid; RPR, rapid plasma reagin; TPPA, *T. pallidum* particle agglutination.*

### Data Processing and Quality Control of Untargeted Metabolomics Analysis

**FIGURE 1:**
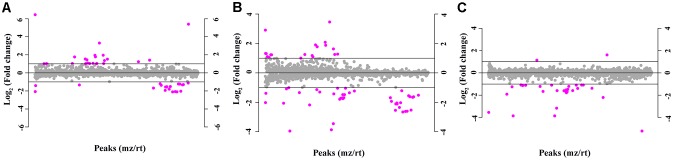
The molecular features of 1,808 metabolite components based on the normalized peak intensity. Purple dots indicate metabolites with a fold change greater than 2, while the gray dots indicate the remaining metabolites. **(A)** Neurosyphilis patients vs. syphilis/non-neurosyphilis patients; **(B)** neurosyphilis patients vs. syphilis-free patients; **(C)** syphilis/non-neurosyphilis patients vs. syphilis-free patients.

**FIGURE 2:**
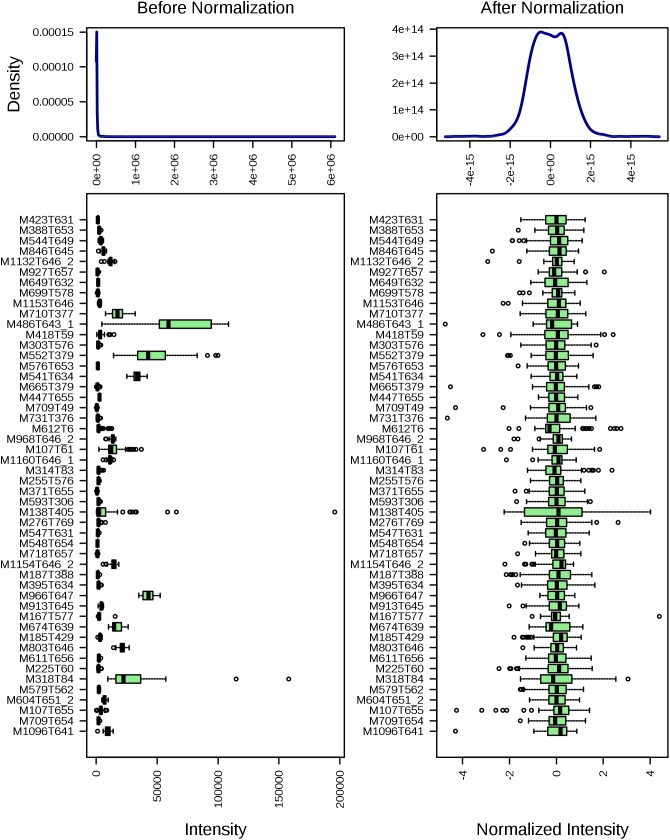
The distribution of input data values before (left) and after (right) normalization.

**FIGURE 3:**
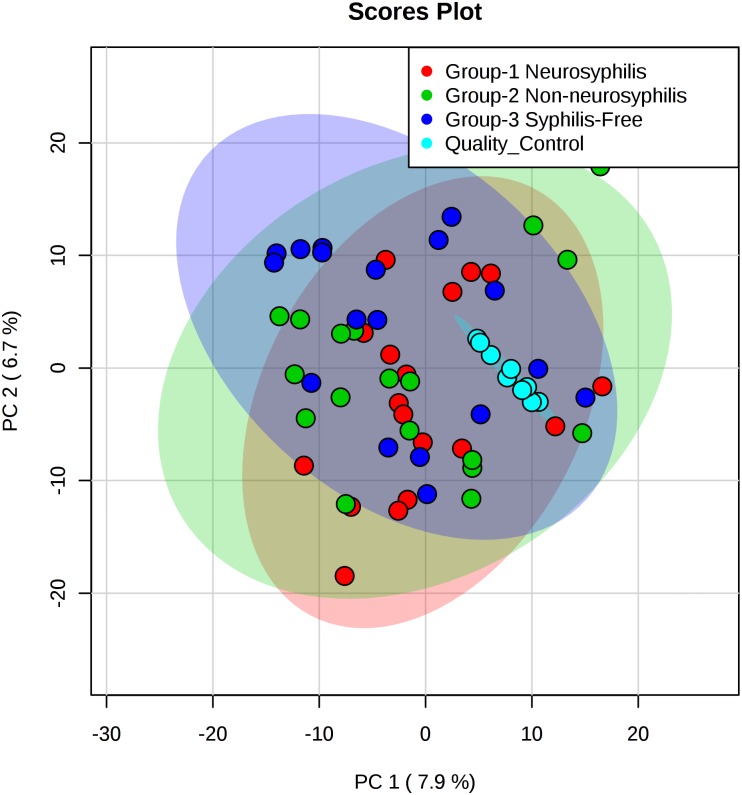
PCA score plots based on the UHPLC-Q-TOF/MS data of CSF samples. Light red oval represents the 95% CI of score calculated from each neurosyphilis patient. Light green oval represents the 95% CI of score calculated from each syphilis/non-neurosyphilis patient. Light blue oval represents the 95% CI of score calculated from syphilis-free patient.

The total ion chromatogram of the quality control sample showed that the overlaps of the spectral peak of the QC samples were within slight changes, suggesting that the method has good reproducibility overall. A total of 1,808 molecular features were extracted from each sample using XCMS software, and the peak intensity of each feature was obtained (Figure 1). Before the subsequent analysis, the data were subjected to a data integrity check, and no missing values were detected. We applied the log transformation and Pareto scaling to the data, and the metabolomics data presented a normal distribution after these processes (Figure 2). Principal component analysis (PCA) was carried out using the molecular features of all the groups from the study, including QC samples. The distribution of metabolic profiles for the test samples and QC samples in PCA are shown in Figure 3. All of the QC injections were clustered tightly in the PCA space. The consistency of the repeated QC injections and reliable data quality across all the samples revealed the potency of the method for metabolic profiling studies during the experiment.

### Untargeted Metabolomics Analysis of CSF Obtained From Study Participants

**FIGURE 4:**
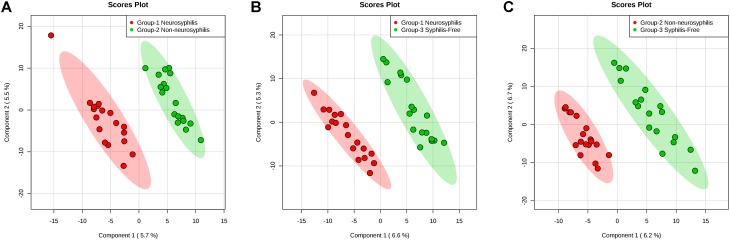
PLS-DA plots based on the UHPLC-Q-TOF/MS data of CSF samples. **(A)** Neurosyphilis patients vs. syphilis/non-neurosyphilis patients (R2 = 0.985, Q2 = 0.5); **(B)** neurosyphilis patients vs. syphilis-free patients (R2 = 0.993, Q2 = 0.5); **(C)** syphilis/non-neurosyphilis patients vs. syphilis-free patients (R2 = 0.986, Q2 = 0.339).

To identify ion peaks that could possibly be used to differentiate the metabolite profiles among the three groups, we established a supervised PLS-DA model that was concentrated on the actual class discriminating variations. In 4A–4C, the multiple comparisons among the three groups all showed clear separation. The goodness of fit (R2) and prediction ability of the model (Q2) by the first three components were 0.985 and 0.5; 0.993 and 0.5; 0.986 and 0.339, respectively, for differentiating neurosyphilis patients and syphilis/non-neurosyphilis patients (Figure 4A); neurosyphilis patients and syphilis-free patients (Figure 4B); and syphilis/non-neurosyphilis patients and syphilis-free patients (Figure 4C).

**FIGURE 5:**
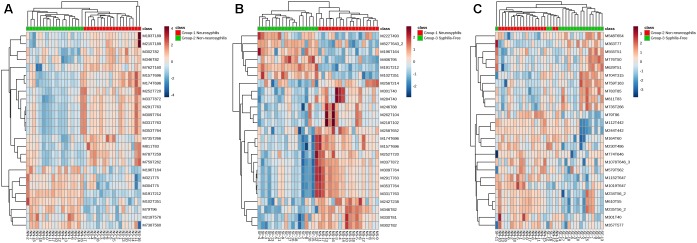
Heatmap of clustering analysis of three groups. **(A)** Neurosyphilis patients vs. syphilis/non-neurosyphilis patients; **(B)** neurosyphilis patients vs. syphilis-free patients; **(C)** syphilis/non-neurosyphilis patients vs. syphilis-free patients.

Based on the VIP calculated by the PLS-DA model, the hierarchical analysis was conducted using Euclidean distance with an average clustering algorithm. Through this analysis and a heat map, the similarity of the metabolite abundance profiles was presented (Figure 5). The results showed a satisfactory discriminatory power between neurosyphilis patients and syphilis/non-neurosyphilis patients (Figure 5A) and between neurosyphilis patients and syphilis-free patients (Figure 5B). For discriminating syphilis/non-neurosyphilis patients and syphilis-free patients, the heat map showed a lower discriminatory power (Figure 5C).

### Identification of Differential CSF Metabolites

We included metabolites with a VIP greater than 1 to conduct Student’s *T*-test to verify whether they are differentially expressed or significantly different in CSF metabolites among neurosyphilis patients, syphilis/non-neurosyphilis patients and syphilis-free patients. A total of 1,808 metabolite components were obtained from each group. Differential metabolite components are described in Table 2 according to the intersection of VIP >1.0 and *P*-value <0.1. The L-gulono-gamma-lactone, D-mannose, and hypoxanthine levels were significantly decreased in neurosyphilis patients compared with syphilis/non-neurosyphilis patients with fold changes of 0.517, 0.872, and 0.745 (*P* < 0.05), respectively. In contrast, an 87.369-fold change in N-acetyl-L-tyrosine levels was observed in neurosyphilis patients compared with syphilis/non-neurosyphilis patients (*P* < 0.05), and an approximately 7.5 times increase was found in neurosyphilis patients compared to syphilis-free patients (0.05 < *P* < 0.1). In addition, significant differences in L-gulono-gamma-lactone were found between the neurosyphilis patients and syphilis-free patients; syphilis/non-neurosyphilis patients and syphilis-free patients had fold changes of 0.646 and 1.591, respectively.

**Table 2:** List of differential CSF metabolites in three groups of study participants.

Comparison	Metabolite	Rt (sec)	m/z	VIP	Fold change	*P*-value
NS vs. Non-NS	L-gulono-gamma-lactone	206.4	196.0808	4.371	0.517	0.024*
	D-mannose	559.8	198.0968	3.142	0.872	0.020*
	N-acetyl-L-tyrosine	69.34	268.0621	5.156	87.369	0.019*
	Hypoxanthine	303.4	137.0447	2.042	0.765	0.002*
NS vs. syphilis-free	L-gulono-gamma-lactone	163.6	196.0808	2.342	0.646	<0.001*
	D-mannose	559.8	198.0968	3.621	0.850	0.061
	N-acetyl -L-tyrosine	69.34	268.0621	4.791	7.492	0.069
NN cases VS NS cases	Hypoxanthine	303.366	137.0447	2.0645	0.765066	0.000692
Non-NS vs. syphilis free	L-gulono-gamma-lactone	206.4	196.0808	4.223	1.591	0.026*
	D-mannose	498.7	198.0966	1.078	0.797	0.071
	N-acetyl -L-tyrosine	69.34	268.0621	2.424	0.086	0.061
	*S*-methyl-5′-thioadenosine	144.7	298.0964	1.421	0.855	0.054
	L-Leucine	485.931	132.1009	1.32648	1.16155	0.071789


*NS, neurosyphilis; Non-NS, syphilis/non-neurosyphilis; Rt, retention time; VIP, variance importance for projection. ^∗^P < 0.05.*

### KEGG Pathway Analysis of Differential CSF Metabolites

**FIGURE 6:**
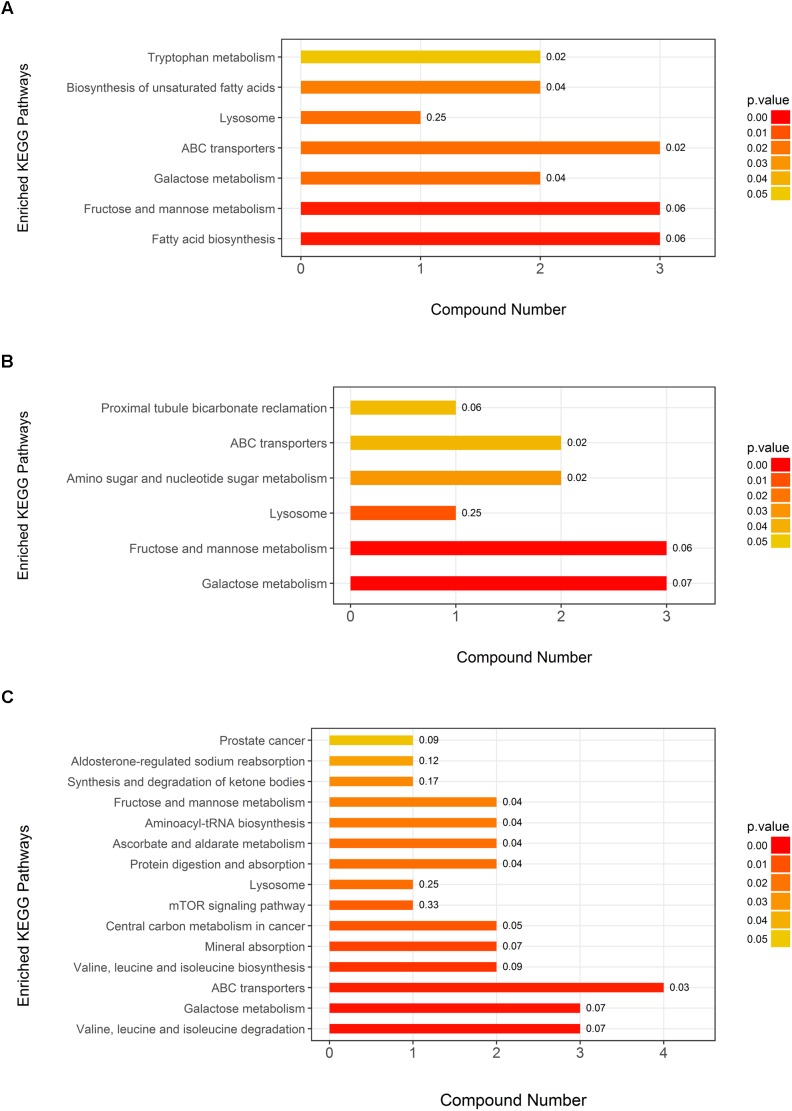
KEGG pathway analysis of the differential metabolite components in the three groups. The *P*-value of each pathway was demonstrated by the color of bar, and the rich factor of each pathway generated by using KEGG analysis was presented in the number after the bar. **(A)** Neurosyphilis patients vs. syphilis/non-neurosyphilis patients; **(B)** neurosyphilis patients vs. syphilis-free patients; **(C)** syphilis/non-neurosyphilis patients vs. syphilis-free patients. The numbers presented in the bar were rich factors generated by using KEGG analysis.

The KEGG pathway analysis showed seven metabolic pathways that were enriched in neurosyphilis patients compared to syphilis/non-neurosyphilis patients; these pathways included tryptophan metabolism, biosynthesis of unsaturated fatty acids, fatty acid biosynthesis, lysosome, ABC transporters, fructose and mannose metabolism, and galactose metabolism (Figure 6A). In a comparison of neurosyphilis patients and syphilis-free patients, there were only six pathways enriched, including proximal tubule bicarbonate reclamation, amino sugar and nucleotide sugar metabolism, lysosome, ABC transporters, fructose and mannose metabolism, and galactose metabolism (Figure 6B). In addition, 15 pathways were identified as enriched pathways when comparing syphilis/non-neurosyphilis patients and syphilis-free patients (Figure 6C). Among them, there were overlapping pathways with the former two comparisons, including fructose and mannose metabolism, lysosome, ABC transporters, and galactose metabolism.

## Discussion

Many studies have demonstrated that pathogens cause distinct host metabolomes, which show involvement of fructose and galactose metabolism, amino acid metabolism, lipid metabolism, and nucleotide metabolism, to adapt to their new environment (Langley et al., 2013; Semmo et al., 2015; Chen et al., 2017; Du et al., 2017). Metabolomics is a powerful tool for studying metabolic processes, identifying crucial biomarkers responsible for metabolic characteristics, and revealing metabolic mechanisms. CSF contains many metabolites, and their variations can offer biochemical clues into central nervous system diseases and possibly provide information on the physiology and diagnostic markers of diseases.

To the best of our knowledge, CSF metabolomics profiles have not previously been described for neurosyphilis. In the present study, we conducted an untargeted metabolomics analysis on CSF collected from neurosyphilis patients to gain a better understanding of the metabolic changes of neurosyphilis patients. We evaluated the experimental process by PLS-DA and hierarchical clustering analysis, and clear separation between groups was found. We verified the differential metabolites using Student’s *T*-test, and several significantly differential metabolites were identified, including L-gulono-gamma-lactone, D-mannose, N-acetyl-L-tyrosine, and hypoxanthine, between neurosyphilis patients and syphilis/non-neurosyphilis patients. The differences in L-gulono-gamma-lactone, D-mannose, and hypoxanthine between neurosyphilis patients and syphilis/non-neurosyphilis patients were less than two times. More specifically, an 87.369-fold change was observed in neurosyphilis patients compared with syphilis/non-neurosyphilis patients, and a 7.492-fold change of N-acetyl-L-tyrosine was observed in neurosyphilis patients compared to syphilis-free patients. Currently, the diagnosis of neurosyphilis relies heavily on serological tests of CSF, such as VDRL and/or TPPA. However, the sensitivity and specificity of serological tests of CSF are not satisfactory based on the previous practices (Jiang et al., 2011). N-acetyl-L-tyrosine may be used as an indicator to distinguish neurosyphilis patients from syphilis/non-neurosyphilis patients. N-acetyl-L-tyrosine is a precursor of the essential neurotransmitter dopamine. Due to the absence of investigations into CSF metabolites in neurosyphilis patients, the role of tyrosine in neurosyphilis remains unknown. Similar to LPS, two purified *T. pallidum* lipoproteins (Tp47 and Tp17) induced NF-kB translocation in THP-1 human monocytoid cells (Norgard et al., 1996). There is considerable evidence for the involvement of tyrosine phosphorylation events (particularly mitogen-activated protein kinases) in LPS signaling and *T. pallidum* lipoproteins (English et al., 1997). However, elevated tyrosine levels may lead to increased formation of dopamine by the combined effects of tyrosine hydroxylase and tryptophan decarboxylase (Sehara et al., 2017). The enhanced level of dopamine would lead to neurobehavioural abnormalities, including increased psychomotor activity, hyperalertness, agitation, irritability, restlessness, combativeness, distractibility, and psychosis (Maldonado, 2008), and some of them overlap with the clinical symptom of neurosyphilis. Moreover, excessive dopamine is capable of inducing neuronal cell death and apoptosis via the caspase-3 pathway (Pedrosa and Soares-da-Silva, 2002). This change could partly explain the higher tyrosine levels in neurosyphilis patients compared to syphilis/non-neurosyphilis patients and syphilis-free patients and the neurobehavioural symptoms caused by *T. pallidum* infection in the central nervous system.

In addition, significantly different L-gulono-gamma-lactone levels were found not only between the neurosyphilis patients and syphilis/non-neurosyphilis patients but also between the neurosyphilis patients and syphilis-free patients, as well as the syphilis/non-neurosyphilis patients and syphilis-free patients. The fold change was less than two. The L-gulono-gamma-lactone levels were significantly decreased in neurosyphilis patients compared with syphilis/non-neurosyphilis patients. L-gulono-gamma-lactone is an immediate precursor of Vitamin C, and its conversion into Vitamin C is catalyzed by L-gulono-1,4-lactone oxidase (Ching et al., 2014). Reduced levels of Vitamin C in the brain may trigger dangerous levels of oxidative stress during normal aging, particularly during inflammatory neurodegenerative diseases (Dixit et al., 2015).

We also observed decreased levels of D-mannose and hypoxanthine in neurosyphilis patients compared with syphilis/non-neurosyphilis patients. The decrease in D-mannose may be due to *T. pallidum*, which is capable of metabolizing mannose as an alternative carbon source under insufficient glucose (Gonzalez et al., 2005). The reason for the decreased hypoxanthine levels in CSF in neurosyphilis patients compared to syphilis/non-neurosyphilis patients and its role in the development of neurosyphilis should be further explored.

We also conducted a metabolomics pathway analysis based on the differentially expressed metabolites identified by employing statistical analysis. Some overlapping pathways, including lysosome, ABC transporters, galactose metabolism and fructose and mannose metabolism, were found in all comparisons. According to the previous literature, *T. pallidum* lacks tricarboxylic acid cycle enzymes and an electron transport chain (Fraser et al., 1998) but does carry enzymes for absorption of amino acids and fatty acids, which indicated that this pathogen has to derive some essential macromolecules from the host. The ABC transporter encoded by the *tro* operon plays an important role in this process and has been identified as an enriched pathway in the present study. Fructose and mannose metabolism was also found to be an enriched pathway. Under the circumstances of insufficient glucose, *T. denticola* is capable of metabolizing mannose as an alternative carbon source in an *in vitro* tissue culture system (Gonzalez et al., 2005). Therefore, *T. pallidum* may take up mannose in the CSF to reproduce itself, especially in a low-glucose environment, such as CSF. In the pathway analysis, most of the overlapping pathways are related to alternative carbon sources of *T. pallidum*.

There are some limitations in the present study. First, the sample size is limited. Second, further validation conducted on a larger population was absent. Whether the same changes take place in other neurological diseases, it need to do some experiments to verify that. Still, several metabolites were associated with neurological complications related to *T. pallidum* infection, and further investigation should be conducted to determine their exact function.

In summary, we conducted a metabolomics study on metabolites of CSF from neurosyphilis patients for the first time. Several significant differences in metabolites were identified, including differences in L-gulono-gamma-lactone, D-mannose, N-acetyl-L-tyrosine, and hypoxanthine. Among them, N-acetyl-L-tyrosine was 87.369 times more common in patients with neurosyphilis than syphilis/non-neurosyphilis patients. These differential metabolites could potentially improve neurosyphilis diagnostics in the future. In addition, the role of these differential metabolites in the development of neurosyphilis deserves further exploration.

## Author Contributions

L-LL, J-HY, and T-CY conceived and designed the experiments. L-LL, WC, M-LT, and H-LZ performed the experiments. YL, XL, and J-JN analyzed the data. WC, YL, and L-RL contributed to reagents, materials, and analysis tools. L-LL wrote the manuscript. J-HY, J-JN, and T-CY revised the manuscript critically for important intellectual content. All authors read and approved the final manuscript.

## Conflict of Interest Statement

The authors declare that the research was conducted in the absence of any commercial or financial relationships that could be construed as a potential conflict of interest.
